# A contemporary reassessment of the enhanced transient expression system based on the tombusviral silencing suppressor protein P19

**DOI:** 10.1111/tpj.16032

**Published:** 2022-12-14

**Authors:** Florence Jay, Florian Brioudes, Olivier Voinnet

**Affiliations:** ^1^ Department of Biology Swiss Federal Institute of Technology (ETH‐Zürich) Universitätstrasse 2 8092 Zürich Switzerland

**Keywords:** transient expression, agro‐infiltration, RNA silencing, P19 protein, siRNAs, technical advance

## Abstract

Transient transgenic expression accelerates pharming and facilitates protein studies in plants. One embodiment of the approach involves leaf infiltration of *Agrobacterium* strains whose T‐DNA is engineered with the gene(s) of interest. However, gene expression during ‘agro‐infiltration’ is intrinsically and universally impeded by the onset of post‐transcriptional gene silencing (PTGS). Nearly 20 years ago, a simple method was developed, whereby co‐expression of the tombusvirus‐encoded P19 protein suppresses PTGS and thus enhances transient gene expression. Yet, how PTGS is activated and suppressed by P19 during the process has remained unclear to date. Here, we address these intertwined questions in a manner also rationalizing how vastly increased protein yields are achieved using a minimal viral replicon as a transient gene expression vector. We also explore, in side‐by‐side analyses, why some proteins do not accumulate to the expected high levels in the assay, despite vastly increased mRNA levels. We validate that enhanced co‐expression of multiple constructs is achieved within the same transformed cells, and illustrate how the P19 system allows rapid protein purification for optimized downstream *in vitro* applications. Finally, we assess the suitability of the P19 system for subcellular localization studies – an originally unanticipated, yet increasingly popular application – and uncover shortcomings of this specific implement. In revisiting the P19 system using contemporary knowledge, this study sheds light onto its *hitherto* poorly understood mechanisms while further illustrating its versatility but also some of its limits.

## Significance Statement

Since its disclosure in 2003, agro‐infiltration used in combination with the P19 RNA silencing suppressor has been a popular method to achieve high levels of transient gene expression in *Nicotiana benthamiana*, though the underlying molecular mechanisms have remained unclear. By shedding light on these mechanisms, the present contemporary reassessment of the method allows improvements to protein yields and further illustrates its versatility, but also exposes and rationalizes some of its limits.

## INTRODUCTION

By respectively promoting nucleotide sequence‐specific chromatin compaction and enhanced mRNA turnover, transcriptional and post‐transcriptional gene silencing (TGS, PTGS) are major impediments to stable transgene expression in plants. Studies of transgene silencing in the model plant *Arabidopsis thaliana* have revealed that both TGS and PTGS are triggered by long double‐stranded RNA (dsRNA) (Beclin et al., 2002; Mourrain et al., 2000; Vaucheret et al., 1998). This molecule is processed into populations of small interfering RNA (siRNA) duplexes by one of four Dicer‐like (DCL) proteins encoded in the Arabidopsis and many other plants' genomes (Bologna & Voinnet, 2014). DCL4 and DCL2 have signature 21‐nt and 22‐nt‐long siRNA products, both of which can promote PTGS upon their incorporation into ARGONAUTE1 (AGO1)‐clade silencing effector proteins. AGO1 uses siRNAs as molecular guides to retrieve sequence‐complementary mRNAs and execute their silencing mainly via endonucleolytic cleavage. DCL3 processes dsRNA into 24‐nt siRNAs, which, loaded into AGO4‐clade members, guide *de novo* RNA‐directed DNA methylation (RdDM) at the siRNA loci of origin (Bologna & Voinnet, 2014). While RdDM mediated by promoter‐derived dsRNA/siRNAs usually results in TGS (Mette et al., 2000), gene body methylation caused by ORF‐derived siRNAs – which often accompanies PTGS – is largely inconsequential to transgene expression (Taochy et al., 2019). At least two non‐mutually exclusive sources of dsRNA have been identified as triggers of transgene silencing in stable transformants, ultimately converging in dsRNA production and downstream processing into siRNAs. In sense‐PTGS (S‐PTGS), aberrant (i.e., uncapped or PolyA^−^) mRNAs ((ab)mRNAs) spawned from presumably highly transcribed transgenes evade RNA quality control (RQC) and are instead competitively *de novo* converted into dsRNA by endogenous RNA‐dependent RNA polymerases (RDRs) (reviewed in Liu & Chen, 2016). In inverted repeat (*IR*)‐PTGS, rearranged transgene arrays, which are common transformation artifacts, form *IRs* whose transcription creates RDR‐independent sources of dsRNA (Beclin et al., 2002; Henderson et al., 2006; Himber et al., 2003). *IR*‐PTGS may also be deliberately achieved for the purpose of experimental gene knockdown via RNA interference (RNAi), using tailored ‘stem‐loop’ transgenes (Himber et al., 2003; Mette et al., 2000).

In Arabidopsis and other plants, both S‐ and *IR*‐PTGS pathways have endogenous gene regulatory counterparts (Henderson et al., 2006; Vaucheret, 2005) operating in complement to, or in conjunction with, a third endogenous PTGS pathway based on microRNAs (miRNAs) (Bologna & Voinnet, 2014). DCL1‐dependent 21‐ to 24‐nt miRNAs are often evolutionary conserved and excised as discrete species from genome‐encoded, imperfect stem‐loop primary transcripts. Incorporated into mostly AGO1, they regulate the abundance/translation of co‐evolving miRNA‐complementary transcripts via PTGS (Voinnet, 2009). Last but not least, mechanisms resembling *IR*‐ and S‐PTGS form the basis of a universal plant antiviral defense system primarily involving DCL4, DCL2, and AGO1/2. The *IR*‐PTGS‐like mechanism uses virus replication‐derived dsRNA to produce 21‐/22‐nt virus‐derived siRNAs (vsiRNAs), while the S‐PTGS‐like mechanism enables their amplification from single‐stranded viral RNA via RDR activities, thereby presumably keeping pace with the mounting viral load (Pumplin & Voinnet, 2013). As a counter‐defensive strategy, most plant viruses have evolved viral suppressors of RNA silencing (VSRs) targeting various and sometimes multiple steps of the plant antiviral silencing response (Pumplin & Voinnet, 2013; Wu et al., 2010). For instance, the tombusviral P19 protein, one of the best biochemically characterized VSRs, forms homodimeric ‘calipers’ structurally poised to bind, with extremely high affinity, the 21‐/22‐nt siRNA products of antiviral DCL4/DCL2 (Silhavy et al., 2002; Vargason et al., 2003; Voinnet et al., 1999). Binding presumably prevents fruitful loading of siRNAs into AGO1/AGO2, and is accompanied by a characteristic 1–2‐nt trimming of siRNA 3′‐ends by an unknown nuclease, a process also manifested on endogenous miRNAs under authentic infections (Kontra et al., 2016) and transgenic P19 expression conditions (Chapman et al., 2004; Iki et al., 2018; Papp et al., 2003).

Although stably transformed plants can be used as bioreactors to yield theoretically high levels of protein production, optimal performances are rarely achieved due to various impediments manifested all along the (trans)gene expression pathway, TGS and PTGS notwithstanding (Kjemtrup et al., 2014; Obembe et al., 2011). These include, but are not restricted to, protein degradation, poor translation, intrinsic mRNA instability, and mediocre transcription due to genomic position effects. Additionally, some proteins of interests might be toxic when constitutively or even conditionally expressed at high levels in stable transformants. Thus, embryo or seedling lethality during transgenesis naturally incurs selection of low‐ to moderate‐expression lines, or simply compromises altogether the viability of harvested tissues. Transient, as opposed to stable, protein expression has emerged as an interesting alternative overcoming at least some of the aforementioned limitations. While many approaches are available (reviewed in Tyurin et al., 2020), probably none exhibits the simplicity, rapidity, and scalability of the procedure whereby suspensions of *Agrobacterium tumefaciens* strains are pressure‐infiltrated, usually with a syringe, into the leaf air space, a procedure known as ‘agro‐infiltration’. In this process, so called ‘disarmed’ T‐DNAs deprived of the tumor‐inducing genes that cause crown gall disease are engineered with the transgene(s) of interest. Upon T‐DNA transfer into plant cells, transgene expression usually peaks between 3 and 5 days post‐infiltration, upon which infiltrated patches can be harvested for molecular studies or protein extraction (Chen et al., 2013). While the method is, in principle, universally applicable, its efficacy is substantially reduced in many Brassicales, including Arabidopsis, owing to an innate immune response triggered by the pathogen‐associated molecular pattern EF‐Tu produced by *Agrobacterium* (Zipfel et al., 2006). Due to its inability, among other Solanales, to perceive EF‐Tu, the wild tobacco relative *Nicotiana benthamiana* has emerged as a plant of choice with which to carry out transient expression via agro‐infiltration. Other advantages of *N. benthamiana* include its non‐demanding and high‐density growth conditions, leaves that are well adapted to pressure‐infiltration, and a reduced cellular protease profile compared to many other plant species (Kjemtrup et al., 2014).

Despite its clear advantages over stable transformation for pharming and other applications, agro‐infiltration in *N. benthamiana* leaves is almost invariably accompanied by a PTGS response diagnosed by accumulation of transgene‐derived siRNAs in the infiltrated tissues, paralleling transgene expression (Hamilton et al., 2002; Voinnet et al., 2000). In a search for a pragmatic remedy to this problem, it was realized that co‐infiltration with a second *Agrobacterium* strain engineered to transiently express a VSR reduces transgene PTGS and, hence, stabilizes transient expression (Hamilton et al., 2002). The approach was found particularly effective with the tombusviral P19 protein evoked here, granting not only stabilized but also, in many – albeit not all – cases, substantially enhanced expression (Voinnet et al., 2003). Due to its simplicity and efficacy, the enhanced transient expression system has been and remains widely employed by the plant community as well as commercially, with the co‐infiltrated P19 *Agrobacterium* strain often used as a default, as opposed to optional, setting in the procedure (Sainsbury & Lomonossoff, 2014). Despite this success, many questions have remained unanswered, not least what mechanisms underpin siRNA accumulation in agro‐infiltrated tissues, that impede transgene expression and, as a corollary, how P19 suppresses them. Indeed, while a plethora of TGS/PTGS mutants are available in Arabidopsis (Bologna & Voinnet, 2014), studying silencing mechanisms is much harder in the poorly genetically amenable species *N. benthamiana*.

Here, we revisit the P19‐enhanced transient expression system using fundamental knowledge gained in Arabidopsis to decipher the likely mechanisms of PTGS induction in infiltrated tissues and the molecular bases of their inhibition by P19. A better understanding of these processes helps us rationalize how a virus‐based self‐replicating transgene, poorly efficacious when expressed alone, rapidly achieves, under P19 co‐expression conditions, protein yields far exceeding those already obtained with the conventional embodiment of the method. While the approach enables a high degree of simultaneous expression of multiple constructs within single cells, we show that PTGS‐unrelated and protein‐intrinsic properties can negatively impact yield even under conditions of vastly enhanced mRNA accumulation enabled by P19. We further describe how an immuno‐purified enzyme performs substantially better in cell extracts prepared from P19‐co‐infiltrated tissues, indicating that high protein levels achieved with the method *in vivo* translate into high functionality *in vitro*. Last but not least, we assess not only the advantages but also the limits of the P19‐enhanced transient expression system for subcellular localization studies, a widespread yet so far largely non‐scrutinized application of the system. With this contemporary reassessment of the P19 system, we formulate both recommendations and cautions for its optimal use and performances in a variety of settings and applications, and discuss possible further improvements.

## RESULTS AND DISCUSSION

### 21‐nt and 24‐nt siRNAs do not accumulate proportionally and are distinctively affected by P19 over time under non‐saturating transient gene expression conditions

Previous studies have shown how *Agrobacterium*‐mediated transient expression of reporter genes such as the endoplasmic reticulum (ER)‐targeted GFP5 allele (Angell & Page, 2002; Haseloff et al., 1997) is accompanied by the production of 21‐nt and 24‐nt siRNAs (Hamilton et al., 2002; Himber et al., 2003), the cognate products of plant DCL4 and DCL3 (Henderson et al., 2006). Certain VSRs co‐expressed in the transient assay impeded accumulation of these species and some displayed siRNA‐size‐selective effects demonstrating that 21‐nt siRNAs are both necessary and sufficient to mediate PTGS as a major impediment to transient expression (Hamilton et al., 2002; Himber et al., 2003). Among these VSRs, the tombusviral P19 protein exhibits potent effects, in agreement with its ability to suppress transgene, viral, and endogenous PTGS by forming homodimers with high and selective affinity for 21–22‐nt small (s)RNAs, including viral/endogenous siRNAs (Kontra et al., 2016) and endogenous miRNAs (Chapman et al., 2004; Iki et al., 2018; Vargason et al., 2003). Beside the efficacy of the P19 co‐expression approach, how siRNA accumulation and PTGS are triggered during *Agrobacterium*‐mediated transient expression and how, mechanistically, co‐expressed P19 impairs these processes have remained largely elusive.

**Figure 1:**
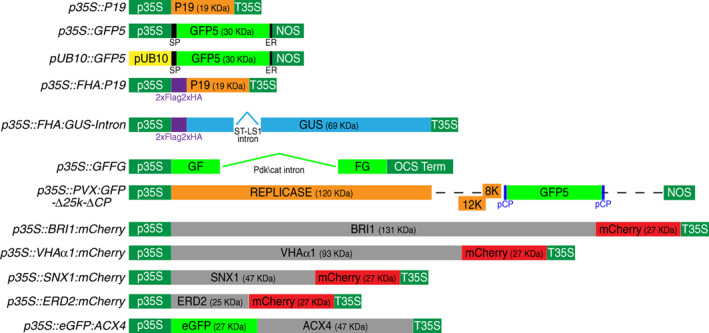
Schematics of the various constructs used in this study. p35S: 35S promoter from Cauliflower mosaic virus (CaMV); pUB10: Arabidopsis *UBIQUITIN10* promoter; T35S: 35S terminator from CaMV; OCS Term: octopine synthase terminator; NOS: nopaline synthase terminator; pCP: coat protein promoter; SP: basic chitinase B signal peptide; ER: HDEL endoplasmic reticulum retention signal.

**Figure 2:**
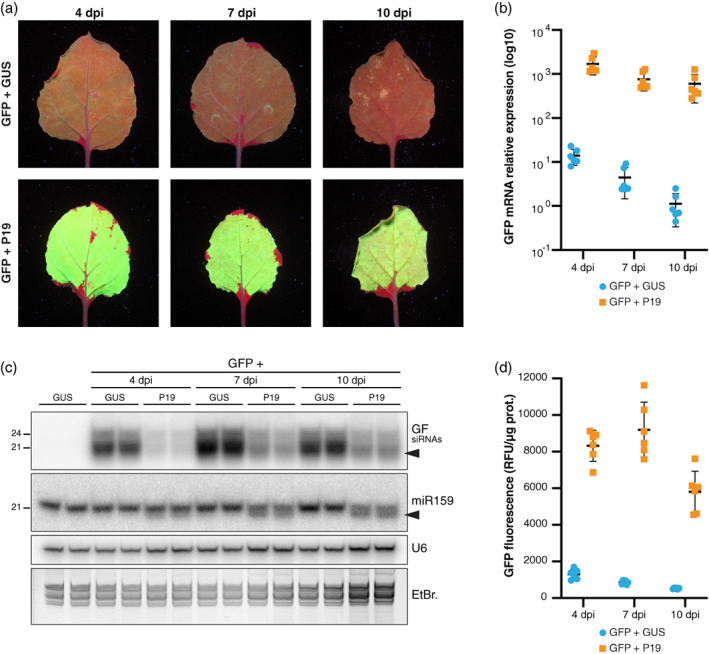
Transient P19 co‐expression dramatically enhances GFP5 mRNA accumulation and reduces 21‐nt siRNA production in infiltrated patches. (a) Images of *Nicotiana benthamiana* leaves, representative of six independent experiments, taken under UV illumination at 4, 7, and 10 days post‐infiltration (dpi) with the constructs *p35S::GFP5* (GFP), *p35S::FHA:GUS‐Intron* (GUS), or *p35S::P19* (P19). Note that chlorophyll fluoresces red under UV. (b) Log_10_‐transformed relative *GFP* mRNA levels in samples depicted in (a) as quantified by RT‐qPCR. Black bars: mean. Error bars: standard deviations. *n* = 6. (c) *GF* siRNA levels in biological duplicates of samples depicted in (a) as analyzed by Northern blot using a probe corresponding to the 5′ part of the *GFP* ORF (‘GF’). The probed miR159 and U6 small RNA were used as an endogenous P19 cargo and RNA loading control, respectively. EtBr.: Ethidium bromide staining provides an additional RNA loading control. Black arrows indicate 3′‐end‐trimmed sRNA species. The experiment was independently repeated three times with similar results. (d) Relative GFP fluorescence units (RFU) measured from the samples depicted in (a). Black bars: mean. Error bars: standard deviations. *n* = 6.

To better dissect the molecular underpinnings of PTGS onset during transient expression and its suppression by P19, we revisited the GFP5‐P19 co‐expression assay in a time‐course analysis involving diluted (OD = 0.3 for each construct for a final OD = 0.6) as opposed to saturated *Agrobacterium* suspensions used previously (Hamilton et al., 2002, Himber et al., 2003). *Nicotiana benthamiana* leaves were infiltrated with an *Agrobacterium* strain carrying a disarmed T‐DNA engineered to express, under the control of the strong and ubiquitous *p35S* promoter, the open reading frame (ORF) of GFP5 (*p35S::GFP5*; Figure 1; Hamilton et al., 2002, Himber et al., 2003). Use of *GFP5* enables measurements of not only mRNA/protein production, but also protein activity monitored visually and non‐invasively *in planta* with a handheld UV lamp and quantified with a fluorescence reader. The diluted *p35S::GFP5 Agrobacterium* strain was mixed 1:1 with a second strain engineered to express either an epitope‐tagged and intron‐containing GUS ORF or the P19 ORF under the control of the *35S* promoter (*p35S::FHA:GUS‐Intron* or *p35S::P19*, respectively; Figure 1). Co‐expressing *p35S::GFP5* with *p35S::FHA:GUS‐Intron* yielded a green fluorescence signal that progressively decreased in intensity from 4 days post‐infiltration (dpi) onward; it was lower at 7 dpi and nearly below visual detection at 10 dpi when only red fluorescence from chlorophyl remained clearly visible (Figure 2a). Correlating with this decrease in green fluorescence was a steady, approximately one‐order‐of‐magnitude decline in *GFP5* mRNA levels from 4 to 10 dpi, as measured in patches individually collected over multiple independent experiments (Figure 2b). Total low‐molecular‐weight RNA analysis from the same tissues revealed accumulation of discrete 21‐nt and 24‐nt *GFP5*‐derived siRNAs reaching an accumulation peak at 7 dpi. At all three time points, however, 21‐nt‐long siRNAs accumulated at higher levels than the 24‐nt‐long species under the non‐saturating transient gene expression conditions used (Figure 2c). These results imply that large amounts of *GFP5*‐derived long dsRNA accumulate within agro‐infiltrated patches because dsRNA is the substrate used by Dicer proteins to produce siRNAs. They also suggest that the dsRNA molecules accounting for accumulation of more abundant 21‐nt siRNAs might be distinct from those underlying the production of 24‐nt siRNAs, reflecting, perhaps, the involvement of distinct dsRNA biosynthetic pathways.

In experiments conducted side‐by‐side, co‐expression of *p35S::GFP5* with *p35S::P19* vastly enhanced green fluorescence compared to co‐expression of *p35S::GFP5* with *p35S::FHA:GUS‐Intron* (Figure 2a). This enhancement, moreover, persisted along the entire 4–10‐day observation time frame, in sharp contrast to the decline in fluorescence observed upon co‐expression of *p35S::GFP5* with *p35S::FHA:GUS‐Intron*. Accordingly, measurements made at 4, 7, and 10 dpi in leaf patches individually collected over multiple independent experiments indicated that, at each time point, the GFP5 fluorescence levels were approximately one order of magnitude higher under *p35S::P19* compared to *p35S::FHA:GUS‐Intron* co‐expression conditions (Figure 2d, confirmed independently by leaf fluorescence scanning in Figure S1). The *GFP5* mRNA levels under *p35S::P19* co‐expression conditions were up to three orders of magnitude (i.e., approximately 1000‐fold) higher than those under *p35S::FHA:GUS‐Intron* co‐expression conditions. Moreover, these mRNA levels remained extremely high over the 4–10‐day observation/sampling period (Figure 2b). Under *p35S::P19* co‐expression conditions, the accumulation of *GFP5*‐derived siRNAs was strongly reduced at 4, 7, and 10 dpi and disproportionally affected the abundant 21‐nt siRNA species. Moreover, a portion of the 21‐nt *GFP5* siRNAs remaining at 7 and 10 dpi showed increasing signs of trimming diagnosed by their enhanced electrophoretic mobility (Figure 2c); 3′‐end 1–2‐nt trimming has been ascribed to an as‐yet unidentified cellular exonuclease acting on the P19‐bound fraction of sRNAs (Iki et al., 2018; Kontra et al., 2016; Papp et al., 2003). An increasing proportion of the 21‐nt miR159, used as a representative of endogenous miRNAs, also displayed trimming over time, suggesting that transiently expressed P19 also binds these molecules as reported in stable transformants. Unlike those of the *GFP5* siRNAs, however, the miR159 steady‐state levels were not overtly decreased. Similar observations on *GFP5* siRNAs and miR159 were made when *p35S* from *p35S::GFP5* was swapped for the Arabidopsis endogenous *UBIQUITIN10* promoter (*pUB10*), which is also known to display strong and constitutive activity (Norris et al., 1993). When co‐agro‐infiltrated with *p35S::FHA:GUS‐Intron*, *pUB10::GFP5* expression yielded a weak green fluorescence signal at 7 dpi similar in intensity to that yielded by co‐infiltrating *p35S::GFP5* with *p35S::FHA:GUS‐Intron* (Figure S2a,b). Moreover, 21‐ and 24‐nt *GFP5* siRNAs also accumulated under *pUB10::GFP5* co‐expression (Figure S2c), suggesting that RNA silencing is a general response to transient transgene expression, regardless of *p35S* usage. Co‐expressing P19 with *pUB10::GFP5* resulted in vastly enhanced green fluorescence (Figure S2a,b), correlating with a strong decrease in *GFP5* siRNA accumulation and evidence of their trimming alongside that of endogenous miR159 (Figure S2c).

That P19 causes a net decrease in transgene‐derived siRNA but not endogenous miRNA levels in both systems suggests either that the protein acts differently on the former versus latter pool of sRNAs in agro‐infiltrated tissues, or that distinct silencing mechanisms involved in the biogenesis of these species influence the net output of P19 binding to sRNAs during transient gene expression.

### The 21‐nt species are mainly secondary siRNAs produced by RDR6 via sense‐PTGS

**Figure 3:**
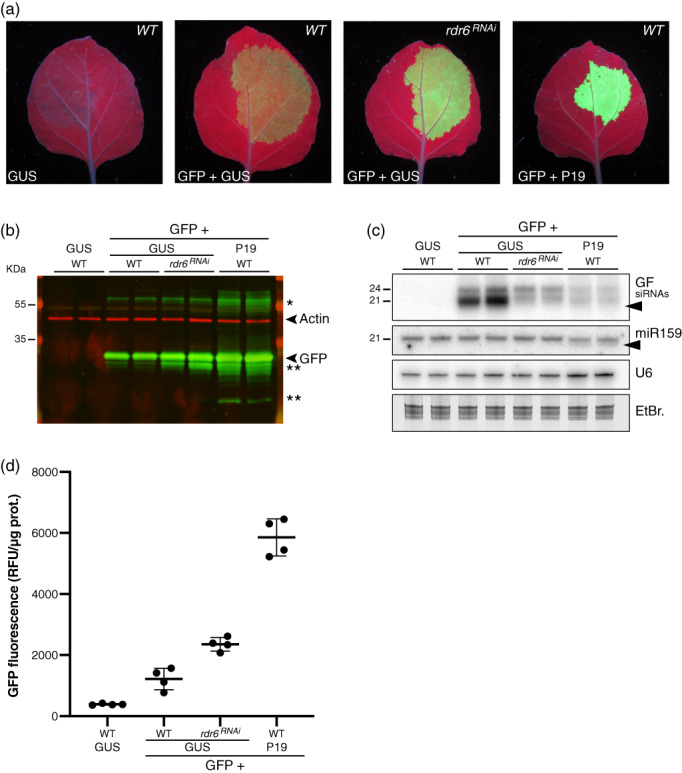
Compared effects of P19 transient co‐expression versus RNAi‐mediated knockdown of NbRDR6 on GFP5 expression and siRNA accumulation in infiltrated patches. (a) Images of *Nicotiana benthamiana* leaves in the WT or the *rdr6* ^*RNAi*^ background, representative of six independent experiments, at 4 dpi with the constructs *p35S::GFP5* (GFP), *p35S::FHA:GUS‐Intron* (GUS), and *p35S::P19* (P19), as indicated, under UV illumination. (b) GFP protein accumulation in biological duplicates of the samples depicted in (a) analyzed by Western blot using an anti‐GFP antibody (green signal). An anti‐actin antibody was used as a control for equal protein loading (red signal). *Indicates higher‐molecular‐weight forms of the GFP protein or potential concatemers thereof. **Indicates truncated byproducts of the GFP protein. The experiment was independently repeated two times with similar results. (c) *GF* siRNA levels in biological duplicates of samples depicted in (a) analyzed by Northern blot. The probed miR159 and U6 small RNA were used as an endogenous P19 cargo and RNA loading control, respectively. EtBr.: Ethidium bromide staining provides an additional RNA loading control. Black arrows indicate 3′‐end‐trimmed sRNA species. The experiment was independently repeated two times with similar results. (d) Relative GFP Fluorescence Units (RFU) measured from samples depicted in (a). Black bars: mean. Error bars: standard deviations. *n* = 4.

No overt feature predisposes the transiently expressed *p35S::GFP5* construct to strong siRNA production. This led us to consider that silencing accompanying agro‐infiltration might be akin, at least partly, to S‐PTGS elicited by sense‐transgenes upon stable, as opposed to transient, transformation. As evoked in the present study's introduction, a key feature of S‐PTGS is its strong dependency upon RDR6 activity, where the dsRNA products are processed by mainly DCL4 into 21‐nt siRNAs (Mourrain et al., 2000; Taochy et al., 2019). To test this idea, we used an established transgenic *N. benthamiana* line in which *Nb*RDR6's activity is constitutively dampened by RNAi (*rdr6* ^*RNAi*^ line; Schwach et al., 2005). In multiple independent experiments, co‐expressing *p35S::GFP5* with *p35S::FHA:GUS‐Intron* resulted in consistently more intense GFP5 fluorescence at 4 dpi (quantified average of approximately 1.9‐fold gain) in leaves with the *rdr6* ^*RNAi*^ compared with the wild‐type (WT) background (Figure 3a,b,d). This enhanced fluorescence was paralleled by a strong and selective reduction, albeit not elimination, of the abundant 21‐nt siRNA species accumulating in the assay, suggesting that silencing triggered during transient gene expression is indeed mostly a manifestation of RDR6‐dependent S‐PTGS (Figure 3c).

We anticipate that the two main and non‐exclusive pathways underpinning S‐PTGS activation upon stable plant transformation are also at work during transient expression via agro‐infiltration in a manner likely explaining the observed siRNA patterns. Firstly, (i) given the sheer levels of transgene expression potentially achievable by this method (as revealed under P19 co‐expression conditions; Figure 2b), elevated amounts of (ab)mRNAs are likely produced. By overwhelming RQC in infiltrated tissues (Gazzani et al., 2004; Moreno et al., 2013; Parent et al., 2015; reviewed in Liu & Chen, 2016), this excess of (ab)mRNA might spontaneously undergo dsRNA conversion by RDRs followed by 21‐nt siRNA production by RDR6‐coupled DCL4 in the cytosol, as previously reported (Mourrain et al., 2000, Taochy et al., 2019). Their 24‐nt counterpart could result from RDR2‐coupled DCL3 activity in the nucleus (Jauvion et al., 2012). Secondly, (ii) rearranged T‐DNA arrays are likely formed during transient expression, such that overlapping sense‐antisense and/or *IR* transcription would constitute RDR‐independent sources of dsRNA processed into 21‐nt and 24‐nt primary siRNAs, as reported during *IR*‐PTGS (Beclin et al., 2002; Henderson et al., 2006; Himber et al., 2003). In either scenario, the 21‐nt fraction of *neo*‐processed primary siRNAs would initiate PTGS of the main bulk of normal, sense‐transgene transcripts, with their breakdown products serving, upon their conversion into long dsRNAs by cytosolic RDR6, as further major sources of DCL4‐dependent 21‐nt secondary siRNAs. This second and prominent RDR6‐amplified phase likely underpins the disproportionate accumulation of 21‐nt versus 24‐nt siRNAs during S‐PTGS in stable transgenic lines (Taochy et al., 2019), as indeed also observed here during transient expression of *p35S::GFP5* (Figures 2c and 3c).

The remaining 21‐nt *GFP5* siRNAs observed upon *p35S::GFP5* expression in *rdr6* ^*RNAi*^ leaves are therefore likely RDR‐independent primary siRNAs formed according to scenario (ii) evoked above, although incomplete RNAi‐mediated knockdown or redundant RDR activities may also be involved. Regardless of their possible origin, these residual 21‐nt siRNAs likely display potent PTGS activity. Indeed, in side‐by‐side analyses, enhanced fluorescence was substantially less pronounced upon co‐expression of *p35S::GFP5* with *p35S::FHA:GUS‐Intron* in *rdr6* ^*RNAi*^ leaves than it was upon its co‐expression with *p35S::P19* in WT leaves (quantified average of approximately 1.9‐fold versus 4.8‐fold gain) despite similar levels of *GFP5* siRNAs remaining under each condition (Figure 3a–d). The key difference in silencing suppression efficacy probably reflects that the 21‐nt *GFP5* siRNAs remaining in *rdr6* ^*RNAi*^ leaves are PTGS‐proficient, whereas those remaining under *p35S::P19* co‐expression are bound and inactivated by P19 as suggested by their enhanced electrophoretic mobility reflecting their likely 1–2‐nt trimming, a feature absent from the *GFP5* siRNAs remaining in *rdr6* ^*RNAi*^ tissues (Figure 3c). If the bulk of *GFP5* siRNAs is mostly composed of RDR6‐dependent secondary molecules, as expected from S‐PTGS, P19‐mediated binding and inactivation of the primary siRNAs required for secondary siRNA amplification would explain the drastic net reduction in *GFP5* siRNA levels observed in infiltrated tissues (Figures 2c and 3c). This would also explain why, in contrast, miR159 levels were not overtly altered by its binding to P19 because miRNA biogenesis does not involve RDR6 or sRNA amplification (Figures 2c and 3c; Voinnet, 2009).

### P19 binding to primary siRNAs likely inhibits amplified S‐PTGS initiated by these molecules

**Figure 4:**
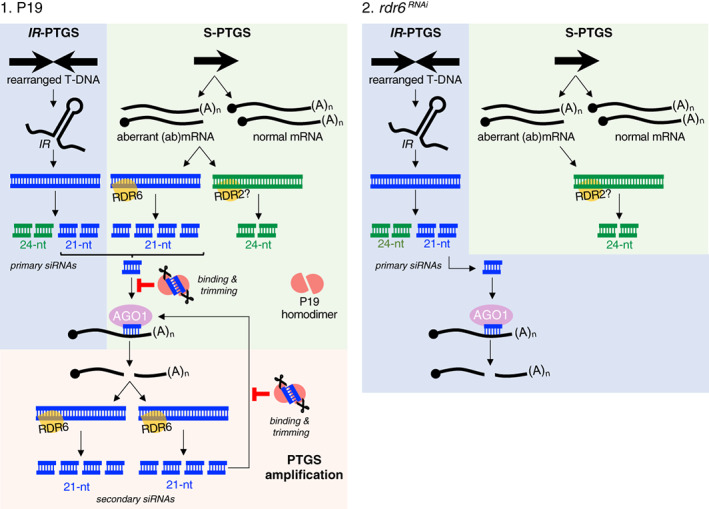
Proposed model for the multilayered action of P19 (scenario 1) during transient gene expression in *Nicotiana benthamiana* leaves as compared to the effect of RDR6 knockdown (scenario 2) using the *rdr6* ^*RNAi*^ background. In scenario 1, *IR*‐PTGS involves transgene arrays organized as inverted repeats (*IRs*). Upon transcription, these form RDR‐independent sources of dsRNA, which is processed into 21‐nt and 24‐nt siRNA species. An additional source of dsRNA is provided through S‐PTGS via RDR6 (leading to 21‐nt siRNA production) and possibly RDR2 (leading to 24‐nt siRNA production), using sense polyA^−^ and uncapped (collectively referred to as to ‘aberrant’) RNAs as templates. 21‐nt siRNAs produced by both *IR*‐ and S‐PTGS are then loaded into AGO1 to guide endocleavage of complementary sense transgene mRNAs. The ensuing polyA^−^ and uncapped RNA breakdown products being aberrant, they serve, in turn, as novel sources of RDR6‐dependent dsRNA and secondary 21‐nt siRNAs. This RDR6‐amplified phase would contribute the main siRNA bulk in infiltrated tissues, explaining the disproportionally high representation of 21‐nt RNAs. Co‐expressed P19 binds to and causes trimming of both primary and secondary 21‐nt siRNAs. Sequestration of the former, in particular, impedes PTGS amplification by RDR6, resulting in a substantial decrease of the dominant 21 nt siRNA fraction. The 24‐nt siRNA levels remain largely unchanged because they are not bound by P19 and, hence, not trimmed. In scenario 2, reduced RDR6 activity in the *rdr6* ^*RNAi*^ background impedes the onset of amplified PTGS, leading to mostly primary (i.e., non‐amplified) 21‐nt siRNA accumulation. The levels of RDR2‐dependent 24‐nt siRNAs presumably accompanying S‐PTGS remain unchanged. While considerably reduced in levels as in scenario 1, the 21‐siRNAs would not undergo trimming.

**Figure 5:**
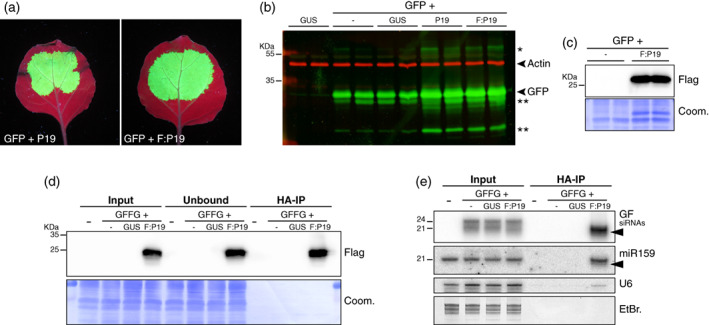
P19 selectively binds the 21‐nt siRNAs produced in infiltrated patches during *IR*‐PTGS. (a) Images of *Nicotiana benthamiana* leaves, representative of six independent experiments, at 4 dpi with the constructs *p35S::GFP5* (GFP), *p35S::P19* (P19), and *p35S::FHA:P19* (F:P19), as indicated, under UV illumination. (b) GFP protein accumulation (green signal) in biological duplicates of the samples depicted in (a) and their controls (*p35S::FHA:GUS‐Intron*: GUS), analyzed by Western blot. An anti‐actin antibody was used to provide a control for equal protein loading (red signal). *Indicates higher‐molecular‐weight forms of the GFP protein or potential concatemers thereof. **Indicates truncated byproducts of the GFP protein. The experiment was independently repeated three times with similar results. (c) FHA:P19 protein levels in biological duplicates of one of the samples depicted in (a) and its controls analyzed by Western blot. Coomassie staining of total proteins (Coom.) provides a control for equal loading. The experiment was independently repeated three times with similar results. (d) FHA:P19 protein levels in the input, unbound fraction, and IP fraction of *N. benthamiana* leaves at 4 dpi with infiltration medium (−), *p35S::FHA:GUS‐Intron* (GUS), *p35S::FHA:P19* (F:P19), and *p35S::GFFG* (GFFG), as indicated. Coom.: as in (c). The experiment was independently repeated four times with similar results. (e) *GF* siRNAs levels in samples depicted in (d) analyzed by Northern blot. miR159 and U6 small RNA were used as an endogenous P19 cargo and RNA loading control, respectively. EtBr.: Ethidium bromide staining provides an additional RNA loading control. Black arrows indicate 3′‐end‐trimmed sRNA species. The experiment was independently repeated four times with similar results.

The model in Figure 4 predicts that, unlike secondary siRNAs, primary siRNAs should not display reduced steady‐state accumulation, being predictably merely sequestered and thereby inactivated by P19. To test this hypothesis, we used an *Agrobacterium* strain expressing, under the control of the *p35S* promoter, an *IR* transgene encompassing the ‘*GF*’ portion of the *GFP5* sequence (*p35S::GFFG*; Figure 1). As shown previously, the *GF‐FG IR* is genetically conditioned to produce dsRNA and, as such, spawns primary siRNAs in an RDR6‐independent manner (Himber et al., 2003). To test the binding of P19 to *GF* primary siRNAs in the absence of an immunoprecipitation (IP)‐graded antibody, we used an N‐terminal FLAG‐HA‐epitope‐tagged allele of the protein expressed under the control of the *p35S* promoter (*p35S::FHA:P19*; Figure 1). We verified that *p35S::FHA:P19* is as efficient as *p35S::P19* in suppressing *GFP5* silencing in the co‐infiltration assay and that FHA:P19 can be robustly immunoprecipitated via anti‐HA IP used to assay binding (Figure 5a–d; Figure S3a). As previously reported in stably transformed WT and *rdr6* Arabidopsis (Himber et al., 2003), transient expression of *p35S::GFFG* spawned, at 4 dpi, near‐equal amounts of DCL4‐dependent 21‐nt and DCL3‐dependent 24‐nt *GF* siRNAs, the cognate pattern of RDR6‐independent primary siRNA production from *IRs* (Beclin et al., 2002, Himber et al., 2003, Henderson et al., 2006; Figure 5e; Figure S3b). This pattern contrasted with the disproportionate levels of 21‐nt species accumulating during transient expression of *p35S::GFP5* presumably as a consequence of their RDR6‐mediated amplification during S‐PTGS (Figures 2c and 3c). Co‐expressing *p35S::GFFG* with *p35S::FHA:P19* did not cause any overt decrease in either *GF* siRNA species' levels compared to *p35S::GFFG* alone or in combination with *p35S::FHA:GUS‐Intron*, a further contrast to the strong reduction in siRNA levels incurred by co‐expressing *p35S::P19* with *p35S::GFP5* (Figures 2c, 3c, and 5e; Figure S3b). The only distinctive feature of the *p35S::FHA:P19 + p35S::GFFG* co‐expression conditions was the enhanced electrophoretic mobility of a fraction of the 21‐nt‐, but not 24‐nt‐, *GF* siRNAs likely reflecting their preferential binding by P19 and the linked 1–2‐nt trimming process (Figure 5e; Figure S3b). Directly supporting this idea, only the 21‐nt *GF* siRNAs were detected in FHA:P19 immune complexes isolated via HA IP, of which a fraction showed signs of trimming as did a fraction of the co‐immunoprecipitated endogenous miR159 (Figure 5e; Figure S3b). Thus, while their binding to P19 causes their trimming (and, presumably, inactivation), it does not reduce the steady‐state levels of primary 21‐nt siRNAs, supporting the proposed model of P19 action (Figure 4).

Collectively, these results suggest that RDR6‐dependent S‐PTGS underpins the bulk of abundant 21‐nt siRNA levels accompanying transient transgene expression and that P19 strongly inhibits this amplified process by binding to and inactivating the 21‐nt primary siRNAs required for its initiation. The 21‐nt *GFP5* siRNAs remaining in tissues co‐expressing *p35S::GFP5* with *p35S::P19* are, therefore, likely mostly composed of P19‐bound and hence, inactive, primary siRNAs.

### Combining P19 expression with that of a recombinant Potato virus X replicon yields further increased GFP5 levels and activity

**Figure 6:**
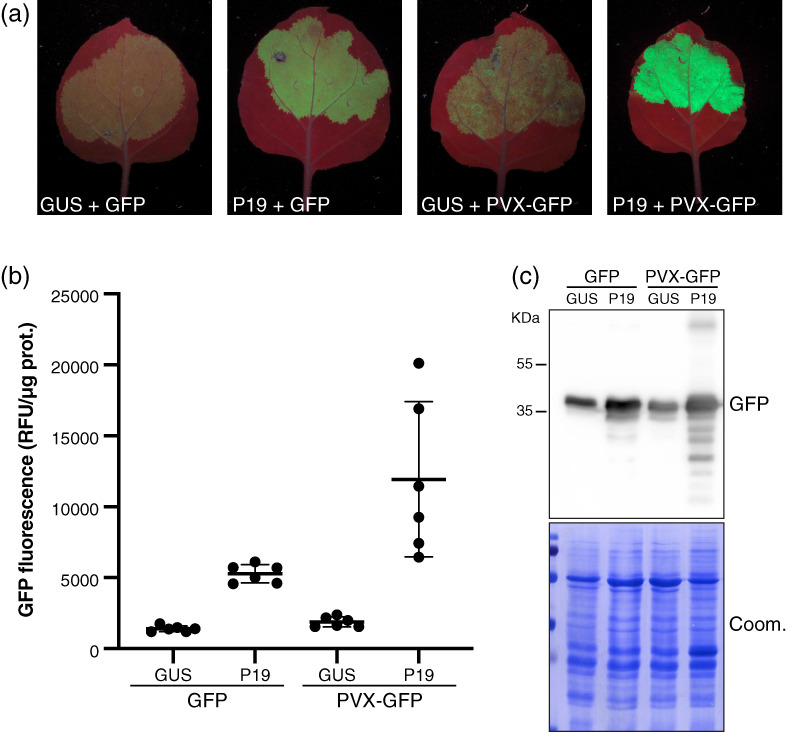
A PVX‐based minimal replicon boosts further GFP5 production in P19 co‐infiltrated patches. (a) Images of *Nicotiana benthamiana* leaves, representative of six independent experiments, at 4 dpi with the constructs *p35S::FHA:GUS‐Intron* (GUS), *p35S::GFP5* (GFP), *p35S::P19* (P19), and *p35S::PVX:Δ25KΔCP:GFP5* (PVX‐GFP), as indicated, under UV illumination. To avoid saturation effects, the OD value of each individual *Agrobacterium* inoculate was 0.1, compared to 0.3 in Figures 2a, 3a, and 5a. (b) Relative GFP fluorescence units (RFU) measured from samples depicted in (a). Black bars: mean. Error bars: standard deviations. *n* = 6. (c) GFP protein accumulation in the samples depicted in (a) analyzed by Western blot. Coomassie staining of total proteins (Coom.) is used as a control for equal loading. The experiment was independently repeated two times with similar results.

The sole known natural function of P19 is to protect the tombusviral RNA against RNA silencing triggered by vsiRNAs spawned during virus replication, which is a source of dsRNA (Vargason et al., 2003). Enhanced replication of recombinant viral vectors via P19 expression has been reported in *N. benthamiana* cell suspension‐ and hairy root‐based settings (Larsen & Curtis, 2012). We thus tested if replication of *Potato virus X* (PVX) would be enhanced by P19 co‐expression in the rapid and easy‐to‐implement *N. benthamiana* leaf agro‐infiltration setting used here. To that effect, we employed *p35S::PVX‐Δ25ΔCP:GFP5* (Voinnet et al., 2000), which is devoid of the 25k and CP ORFs required for viral movement, encapsidation and, hence, disease symptom development (Figure 1). Being innocuous, non‐infectious, and non‐mobile, this near‐minimal PVX replicon is in principle suited to high‐level protein production via *Agrobacterium*‐mediated transient expression without biocontainment issues. The results in Figures 2, 3, and 5 suggested, however, that *p35S::PVX‐Δ25ΔCP:GFP5* would trigger RNA silencing at least at three distinct, yet cumulative, levels. The first level would be akin to RDR6‐dependent S‐PTGS targeting the primary transgene RNA transcribed from the *p35S* promoter, as observed with *p35S::GFP5* (Figures 2a and 3a). The second anticipated level of RNA silencing was that triggered by autonomous replication (and, hence, dsRNA production) of said primary transcripts independently of transgene expression. RNA silencing activated at this level would be conceptually similar to *IR‐*PTGS triggered by *p35S‐GFFG*. A third potential layer of RNA silencing would entail RDR‐dependent dsRNA amplification from replicated single‐stranded viral transcripts or breakdown products thereof, given the established sensitivity of PVX infection to at least RDR6 (Schwach et al., 2005). *p35S::P19* co‐transient expression was predicted to suppress all three RNA silencing layers potentially restricting optimal *GFP5* expression and activity from *p35S::PVX‐Δ25ΔCP:GFP5*. We thus tested if co‐expression of *p35S::P19* with *p35S::PVX‐Δ25ΔCP:GFP5* would yield higher GFP5 levels than co‐expression of *p35S::P19* with the non‐replicative *p35S::GFP5* transgene or co‐expression of the latter with *p35S::FHA:GUS‐Intron* (Figure 6a).

To better quantify these anticipated differences, all *Agrobacterium* strains used in the following comparative experiments were diluted to an OD of 0.1, compared to 0.3 used in Figures 2, 3, and 5. The same UV light intensity and illumination time were used for imaging, however, as were the settings for fluorescence quantification conducted in patches individually collected over multiple independent experiments. In quantitative assessment of GFP5 fluorescence at 4 dpi, the effects of co‐expressing *p35S::FHA:GUS‐Intron* with *p35S::GFP5* or *p35S::PVX‐Δ25ΔCP:GFP5* were similar (Figure 6b). Thus, in the absence of silencing suppression, little to no significant gain was afforded by the replicative nature of the latter owing, presumably, to the full action of the aforementioned, intertwined RNA silencing layers. Co‐expressing *p35S::GFP5* with *p35S::P19* yielded higher GFP5 fluorescence under UV illumination, quantified as an average 3.8‐fold gain, which is lower than that reported in Figures 2, 3, and 5 as expected from the lower bacterial OD used (Figure 6b). However, and despite this suboptimal OD, the gain in GFP5 fluorescence was further increased to 8.5‐fold, on average, by co‐expressing *p35S::PVX‐Δ25ΔCP:GFP5* with *p35S::P19*, resulting in the most intensely bright signal observed among all our GFP5‐based experiments (Figure 6a–c). Thus, the combined transient expression of P19 with a minimal PVX replicon yields expression levels substantially higher than those already achieved by expressing a non‐replicative transgene, either alone or with P19. The results also show that engineering the viral vector itself to co‐express P19 and the gene of interest (Mardanova et al., 2017) is not necessary for this approach to be successful.

### Gains in protein accumulation granted by the P19 system may vary extensively, but are upheld upon expression of multiple, co‐delivered constructs

**Figure 7:**
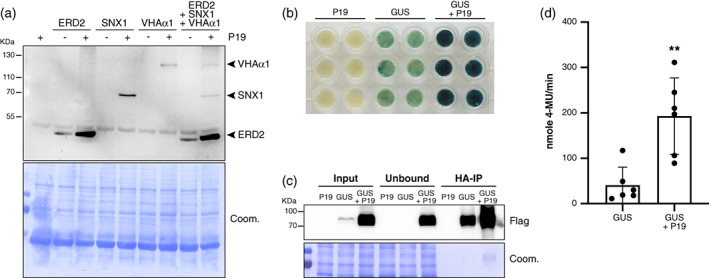
Simultaneous protein co‐expression and enzyme purification using the P19 system. (a) ERD2, SNX1, and VHAα1 protein accumulation in *Nicotiana benthamiana* leaves at 4 dpi with *p35S::P19* (P19), *p35S::ERD2:mCherry* (ERD2), *p35S::SNX1:mCherry* (SNX1), or *p35S::VHAα1:mCherry* (VHAα1), as indicated, analyzed by Western blot using a generic RFP‐specific antibody. Coomassie staining of total proteins (Coom.) was used as a control for equal loading. The experiment was independently repeated two times with similar results. (b) GUS staining of *N. benthamiana* leaf discs at 4 dpi with *p35S::P19* (P19) and *p35S::FHA:GUS‐Intron* (GUS), as indicated. The experiment was independently repeated two times with similar results. (c) FHA:GUS protein accumulation in the input, unbound fraction, and immunoprecipitated (IP) fraction of the samples depicted in (b). Coom.: as in (a). The experiment was independently repeated six times with similar results. (d) GUS activity of the FHA:GUS IP fraction from samples depicted in (b) and (c). Black bars: mean. Error bars: standard deviations. *n* = 6. **P = 0.0087 (Mann–Whitney test).

On the one hand, S‐PTGS is a likely general impediment to *Agrobacterium*‐mediated transient expression, while, on the other, co‐expressed P19 likely broadly prevents this caveat by protecting the mRNAs of interest from degradation. The levels of protein produced from such P19‐protected mRNAs may vary extensively, however. This likely reflects protein‐intrinsic translation efficacies, half‐lives, and, when applicable, varying turnover rates influenced by post‐translational degradation pathways. Distinct biochemical properties of the tested proteins, which ultimately influence the extraction and analytical methods used for their detection, may also artificially contribute to such variations independently of the abovementioned factors. To address this issue comprehensively, we engineered *p35S*‐driven mCherry‐translational fusions to the ORFs of three cellular membranous compartments' markers with dissimilar molecular weights. Hence, the same protocol of insoluble protein extraction and the same generic anti‐RFP antibody could be used in henceforth directly comparable Western blot analyses. Using a bacterial OD of 0.15 for each construct (for a total OD of 0.6, all combined together with *p35S::P19* or *p35S::FHA:GUS‐Intron*), the *cis*‐Golgi marker ERD2 (25 kDa; Jaillais et al., 2008) was detected at 4 dpi without *p35S::P19* co‐expression, while both the endosomal marker SNX1 (47 kDa; Jaillais et al., 2008) and the *trans*‐Golgi network marker VHAα1 (93 kDa; Jaillais et al., 2008) were below detection levels of Western blot analysis (Figures 1 and 7a). Under *p35S::P19* co‐expression conditions, the initial ERD2 levels were substantially increased, whereas SNX1 and VHAα1 became detectable (Figure 7a). These results therefore indicate that the P19 system yields variable gains in otherwise identically extracted and detected proteins exhibiting similar biochemical properties, most likely due to protein‐intrinsic properties upon which P19 is expected to exert little or no effect. This notion is further illustrated by the discrepancy between the extremely high gains in *GFP5* mRNA accumulation granted by P19 co‐expression (as presented in Figure 2b) and those observed at the GFP5 activity (Figure 2d) and protein steady‐state levels (Figures 3b and 5b). Accumulation of low‐molecular‐weight GFP5‐derived fragments revealed by Western blot analysis strongly suggest proteolysis as one possible source of this discrepancy (Figures 3b and 5b). Bands migrating at a higher than expected molecular weight, also detected with the anti‐GFP5 antibody, further suggest the involvement of ubiquitination and 26S proteasome activation (Figures 3b and 5b). Nonetheless, the results with SNX1 and VHAα1 also indicate that P19 can empower detection of transiently expressed proteins that are otherwise too low in abundance to enable their study, let alone purification. Using the rapid *Agrobacterium* leaf infiltration procedure granted, in these two cases, infinite gains in protein production. The results, finally, confirm that the P19 co‐expression effect is indiscriminative of transgene products, presumably because S‐PTGS is a universal impediment intrinsically inherent to the transient expression procedure itself.

We then tested if the above gains in protein production observed in separate agro‐infiltration experiments could be recapitulated if the three membrane markers' *Agrobacterium* strains were co‐infiltrated simultaneously in conjunction with the *p35S::P19* strain. As shown in Figure 7a, this was indeed the case, with the protein gains detected by Western blot analysis being on par with those yielded by separate co‐expression, save slightly lower gains for SNX1. These results are most easily explained if a majority of cells in the co‐infiltrated patch had effectively undergone simultaneous transient transformation by the four T‐DNAs involved. This is consistent with the uniformity of GFP5 expression in infiltrated patches (Figures 2a, 3a, and 5a), the results of Figure 6a, and the previously reported spatial coincidence of reporter genes' expression upon co‐delivery from separate *Agrobacterium* strains (Hamilton et al., 2002; Himber et al., 2003; Voinnet et al., 2000). The ability provided by the P19 system to simultaneously enhance production of multiple proteins within the same cells allows interesting applications in which, for instance, a desirable compound is produced by multiple key enzymes as part of a given biosynthetic pathway.

### The P19 system is suited for high‐level production of recombinant proteins for downstream *in vitro* applications

Increased GFP5 levels are accompanied by increased fluorescence in *p35S::P19* co‐expressing cells, demonstrating that at least part of the over‐accumulated protein is biologically active *in vivo* (Figures 3a,b and 5a,b). Thus, another biotechnological application of the P19 system is to optimize production of recombinant proteins subsequently used for downstream applications upon their purification. For this purpose, the over‐accumulated protein should display high activity levels not only *in vivo* but also *in vitro* after purification. To quantitatively illustrate this additional application of the P19 system, we employed the *p35S::FHA:GUS‐Intron Agrobacterium* strain used so far as a mere negative control in co‐infiltration experiments. The 2×FLAG‐2×HA (FHA)‐tag in the construct enables immuno‐purification of the soluble bacterial β‐glucuronidase (GUS) enzyme, while the presence of an intron prevents its spurious production from *Agrobacterium* cells (Himber et al., 2003). This ensures that FHA:GUS activity measurements exclusively report that of the protein expressed *in planta*. Direct histochemical blue staining of leaf discs isolated from individually collected patches showed, visually, that substantially more FHA:GUS products accumulate upon co‐expression of *p35S::FHA:GUS‐Intron* and *p35S::P19* (Figure 7b). This enhancement correlated with strongly increased FHA:GUS levels accumulating in the co‐infiltrated patches compared with those expressing *p35S::FHA:GUS‐Intron* alone (Figure 7c, Input lanes). Accordingly, more FHA:GUS was HA‐immuno‐purified from leaf patches co‐infiltrated with *p35S::P19* (Figure 7c, HA IP lanes). To test if the enhanced FHA:GUS total enzyme activity observed *in vivo* persisted *in vitro* after purification, immunoprecipitates obtained under either condition were subjected to quantitative *in vitro* MUG assays involving six independent replicates each. These assays measure the hydrolysis rate of 4‐methylumbelliferyl β‐d‐glucuronide (4‐MUG) into 4‐methylumbelliferone (4‐MU), a fluorochrome emitting at 460 nm with negligible substrate background fluorescence (Blazquez, 2007). A 5‐min time‐course analysis revealed that the steady increase in GUS total enzyme activity yielded by FHA:GUS immuno‐purified from patches co‐infiltrated with *p35S::P19* was substantially more pronounced than that yielded by FHA:GUS immuno‐purified from singly infiltrated patches, presumably reflecting the increased yield in FHA:GUS production under P19 co‐expression. Linear regression and use of a standard reference curve showed that an average yield of 193 nmol 4‐MU/min was achieved with the former compared to 40 nmol 4‐MU/min with the latter (Figure 7d; Figure S4). Although the assay displayed some variability, the gain in 4‐MUG production under P19 co‐expression conditions was up to 10‐ or even 20‐fold in some replicates (Figure 7d). These results therefore reinforce the proof of principle that the P19 system can be used to produce higher amounts of active recombinant protein for downstream *in vitro* applications.

### Advantages and limits of the P19 transient expression system for cell biology studies

**Figure 8:**
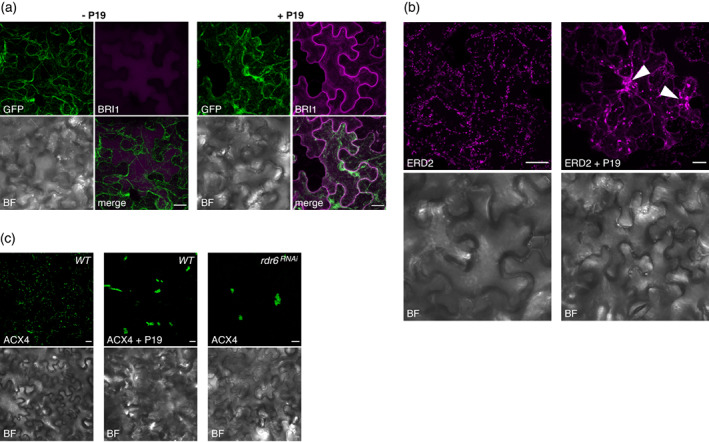
Advantages and limitations in using the transient P19 co‐expression system for subcellular localization studies. (a) Confocal images of *Nicotiana benthamiana* leaves at 4 dpi with *p35S::GFP5* (GFP), *p35S::BRI1:mCherry* (BRI1), and *p35S::P19* (P19), as indicated. (b) Confocal images of *N. benthamiana* leaves at 4 dpi with *p35S::ERD2:mCherry* (ERD2) and *p35S::P19* (P19), as indicated. (c) Confocal images of *N. benthamiana* leaves at 4 dpi with *p35S::eGFP:ACX4* (ACX4) and *p35S::P19* (P19), in the WT or the *rdr6* ^*RNAi*^ background, as indicated. BF in (a–c): Bright field. Scale bars in (a–c): 20 μm. Images are representative of at least three pictures acquired in three (a) or two (b, c) independent experiments.

Possibly the most universal academic use of the *Agrobacterium*‐mediated transient expression system in *N. benthamiana* leaves is as a rapid method for the preliminary assessment of the functionality of transgene constructs. These notably include transcriptional and translational reporter gene fusions destined to *in planta* expression and subcellular localization studies in stable transformants. As shown in Figure 7a with the mCherry fusions to SNX1 and VHAα1, use of P19 as a suppressor of S‐PTGS is imperative, and thereby transformative, in enabling the mere detection of certain proteins of interest. Yet it simultaneously grants visualization of their cellular distribution within infiltrated tissues, including in the jigsaw puzzle‐shaped cells of the leaf epidermis. We present below three case studies conducted with well‐characterized endomembrane markers under the confocal microscope. These were chosen to document distinct outcomes of using P19 in preliminary subcellular localization studies conducted in agro‐infiltrated patches, two of which illustrate some limits of the method for this specific application. In a first experiment, the *p35S::GFP5 Agrobacterium* strain, which produces the ER‐targeted GFP5, was co‐infiltrated with a second strain engineered to express, under the control of the *p35S* promoter, a C‐terminal mCherry translational fusion to the plasma membrane‐associated brassinosteroid receptor BRI1 (Russinova et al., 2004) (*p35S::BRI1:mCherry*; Figure 1). Without co‐infiltrating the *p35S::P19* strain, the green signal yielded by GFP5 was detected in epidermis cells, forming a reticulate network typical of the ER. BRI1::mCherry levels, by contrast, were below the detection limit (Figure 8a, left panels). Co‐infiltrating the two above strains with the *p35S::P19* strain yielded enhanced and readily detectable signals for both *p35S::GFP5* and *p35S::BRI1:mCherry* constructs, with the latter detected, as expected, on the plasma membrane (Figure 8a, right panels). Noteworthily, the enhanced reticulate green and plasma membrane mCherry signals were preponderantly simultaneously detected in epidermal cells. Since P19 is cell autonomous (Brosnan et al., 2019; Devers et al., 2020), as are the membrane‐bound GFP5 and BRI1:mCherry alleles, these observations demonstrate a high incidence of co‐delivery, within single cells, of the three T‐DNAs producing P19, GFP5, and BRI1:mCherry, thus directly supporting the notion already evoked in relation to Figure 7a.

In a separate series of analyses, the mCherry fusion to the *cis*‐Golgi marker ERD2 used in the experiments of Figure 7a was imaged under a confocal microscope. Agreeing with ERD2:mCherry being detectable by Western blot analysis without the need for P19 co‐expression (Figure 7a), a signal highlighting a multitude of Golgi‐derived vesicles was detected when the *p35S::ERD2:mCherry Agrobacterium* strain was delivered alone into *N. benthamiana* leaves (Figure 8b, left panels). A similar, albeit enhanced signal was detected if the *p35S::ERD2:mCherry* strain was co‐delivered with the *35S::P19* strain (Figure 8b, right panels). Unlike in the singly infiltrated tissues, however, several vesicles' aggregates of various sizes also accumulated under the co‐infiltration condition (Figure 8b; arrows). These were likely caused by too high levels of *p35S::ERD2:mCherry* expression as a possible source of cellular stress and/or toxicity; accordingly, the co‐infiltrated leaves showed signs of necrosis observed with neither strain alone (Figure S5). In this case, therefore, use of P19 was not only unnecessary but it yielded, on top of a cognate signal, an undesirable and artifactual signal potentially confounding interpretation of ERD2 subcellular localization.

A third analysis involved an N‐terminal eGFP fusion to the peroxisomal protein ACX4 (Hayashi et al., 1999) expressed under the control of the *p* *35S* promoter (*p35S::eGFP:ACX4*; Figure 1). As seen for *p35S::ERD2:mCherry*, expression of *p35S::eGFP:ACX4* alone was sufficient to yield a cellular signal delineating punctate organelles as expected from the labeling of cognate peroxisomes (Figure 8c, left panels). This pattern was dramatically modified, however, if *p35S::eGFP:ACX4* was co‐expressed with *p35S::P19*, a condition under which the green fluorescence signal delineated large structures presumably reflecting artifactual peroxisomal aggregates (Figure 8c, middle panels). Since peroxisomes have been suggested as subcellular sites of siRNA accumulation (Incarbone et al., 2017), we considered that the high affinity of P19 for these molecules might have caused these aggregates, independently of P19‐mediated PTGS suppression underpinning the visibly enhanced fluorescence signal in co‐infiltrated, compared to singly infiltrated, patches. This was not the case, however, because similar aggregates were observed in singly infiltrated *N. benthamiana* leaves with the *rdr6* ^RNAi^ genetic background, suggesting that such aberrant structures result from mere silencing suppression and, hence, *eGFP::ACX4* over‐accumulation (Figure 8c, right panels). In this particular example, P19 co‐expression was thus not only unnecessary, but it incurred an entirely artificial subcellular localization for ACX4. The latter two examples indicate that P19 co‐expression should not be used as a default setting during transient expression experiments in preliminary cell biology studies, and that singly infiltrated patches – assuming they yield sufficient signal – ought to be systematically inspected in parallel during such applications of the method.

## CONCLUDING REMARKS

This contemporary re‐evaluation of the P19 system, a widely used method developed nearly 20 years ago, now sheds light on the thus far mysterious RNA silencing processes that intrinsically limit *Agrobacterium*‐mediated transient expression in tobacco leaves. Owing to the sheer amount of T‐DNA molecules likely involved in the assay, initial transcription of the transgenes of interest likely generates substantial amounts of (ab)mRNAs that, by overwhelming RQC, stimulate their RDR‐dependent conversion into dsRNA as a source of both 21‐nt (RDR6) and 24‐nt (RDR2) siRNAs. In parallel, T‐DNA rearrangements also likely trigger RDR‐independent *IR*‐PTGS further fueling production of both siRNA species. *A priori*, neither process needs to be activated in every T‐DNA‐transformed cell, since siRNAs are mobile within and outside infiltrated patches (Himber et al., 2003) in a manner that would create RDR substrates upon target mRNA cleavage in virtually all siRNA‐recipient cells (Figure 4). Perhaps counter‐intuitively, therefore, use of *Agrobacterium* suspensions at high ODs –while possibly enhancing initial mRNA transcription – will come at the cost of increased siRNA production as exemplified in previous studies employing saturated bacterial suspensions (Hamilton et al., 2002; Himber et al., 2003; Voinnet et al., 2000). We show here that ODs of approximately 0.1–0.3 will generally constitute a good compromise but, ultimately, optimal protein production (assuming this is the desired outcome) will require empirical adjustments made on a case‐by‐case basis. A transgene encoding the near‐minimal PVX replicon failed, on its own, to boost GFP5 production, presumably because the primary S‐PTGS targeting the non‐replicating *p35S::GFP5* transgene was seconded, in this setting, by the strong and replication‐intrinsic *IR*‐PTGS‐like antiviral response naturally triggered by PVX. By presumably sequestering siRNAs emanating from both sources, P19 enabled protein gains that far exceeded those already obtained with co‐expression of *p35S::GFP5* and *p35S::P19*. Given the universality of the plant RNA silencing‐based antiviral defense system (Lecellier & Voinnet, 2004; Pumplin & Voinnet, 2013), this likely explains why other bio‐contained viral vectors have been successfully used with P19 in the leaf agro‐infiltration procedure (Norkunas et al., 2018), including, for example, in antigen and antibody production (Liu et al., 2005). At least two factors could explain the potency of P19 in circumventing the multi‐layered RNA silencing triggered in agro‐infiltrated tissues. Firstly, being itself expressed from a T‐DNA‐encoded transgene, P19 likely stabilizes its own mRNA in addition to those of co‐delivered constructs. Secondly, the 1–2‐nt trimming of its si/miRNA cargoes likely promotes their release from P19 homodimers, as expected from their crystal structure (Kontra et al., 2016; Vargason et al., 2003). This would allow effective recycling of the VSR, consistent with a multiple‐turnover mode of action previously inferred from *in vitro* experiments and modeling (Rawlings et al., 2011).

Although P19 will broadly stabilize mRNAs of interest, it will remain ineffective in suboptimal downstream steps of the gene expression pathway including poor protein translatability or high turnover rates. This likely explains why great discrepancies might be observed between the gains in mRNA versus protein levels, as exemplified by GFP5 yet to a much lesser extent by GUS. This suggests that the P19 system might be further optimized by engineering mRNAs of interest via codon optimization (Sugio et al., 2010) and/or translation enhancer signals of viral (Carrington & Freed, 1990; Gallie & Walbot, 1992; Jobling & Gehrke, 1987), plant (Agarwal et al., 2014), or artificial (Kanoria & Burma, 2012) origins known to increase transgene expression not only in stable transformants but also during transient expression (Sainsbury & Lomonossoff, 2008). Concomitant inhibition of post‐translational degradation processes such as 26S proteasome‐ or autophagy‐mediated proteolysis using for example drugs (Derrien et al., 2012) or the transient CRISPR/Cas approach (Nekrasov et al., 2013) may further increase protein yield. Efforts to ameliorate the liquid infiltration medium with surfactants and antioxidants or to provide a more conducive ground to gene expression under the cellular stress incurred by the procedure by, for example, modifying the cell cycle or heat shocking samples (Norkunas et al., 2018) also hold great potential. Nonetheless, we also highlighted clear limits of the P19 system in preliminary cell biological studies by showing how artifactual agglomerates – due, presumably, to protein overaccumulation – may confound interpretations depending on the factor under study. We thus recommend that such observations be ultimately validated in stable transgenic lines.

## METHODS

### Plant material and growth conditions

Transgenic *N. benthamiana rdr6* ^*RNAi*^ was previously described in Schwach et al. (2005). Plants were grown on soil at 21°C in 16 h light/8 h dark conditions (light intensity: 120 μE m^−2^ sec^−1^). All agro‐infiltrations were performed on the fourth and fifth leaves of approximately 4 week‐old plants.

### Plasmids and cloning procedures

The *p35S::P19*, *p35S::GFP5*, and *p35S::PVX:GFP‐∆25k‐∆CP* expression vectors were described previously (Angell & Page, 2002; Hamilton et al., 2002; Voinnet et al., 2000). Briefly, removal of a cryptic intron by modifying codon usage in the *GFP* ORF (*Aequorea victoria*) led to mGFP4, also known as *GFP4*; *mGFP4* was then further modified with an N‐terminal Chitin signal peptide and the ER‐targeting HDEL amino acid sequence in the C‐terminal region, leading to *mGFP4‐ER*, also known as *GFP5* (Angell & Page, 2002; Haseloff et al., 1997). The complete sequence of the *p35S::GFP5* expression cassette within the pBI121 binary vector backbone (Haseloff et al., 1997) is shown in Figure S6. Gateway cloning technology (Thermo Fisher Scientific, www.thermofisher.com) was used to assemble the other expression constructs presented in Figure 1. DNA cloning was conducted using Phusion High‐Fidelity DNA Polymerase (Thermo Scientific, www.thermofisher.com) and primers given in Table S1. The FHA:P19‐expressing construct was generated by introducing attB1, attB2, and FHA‐tag sequences into the P19 coding sequence by PCR. The attB‐flanked DNA fragment was BP‐recombined into pDONR221 and the resulting entry vector was LR‐recombined in the destination vector pB7m24GW (Karimi et al., 2007) together with an attL4/attR1 entry vector harboring the *p35S* promoter or the *pUB10* promoter. The FHA:GUS‐Intron expression vector was generated by LR‐recombining entry vectors containing an attL4/attR1‐flanked 35S promoter sequence, an attL1/attL2‐flanked FHA‐tag sequence, and an attR2/attL3‐flanked ST‐LS1‐intron‐containing GUS sequence into the destination vector pB7m34GW (Karimi et al., 2007). The GFFG expression vector was generated by recombining an attL1/attL2 vector containing the first 400 nucleotides of the coding DNA sequence of mGFP6 in the destination vector pBm42GWIWG8 (Karimi et al., 2007). BRI1, VHAα1, SNX1, and ERD2 expression constructs were produced by LR‐recombining the relevant attL1/attL2 entry vectors together with an attL4/attR1 entry vector harboring a 35S promoter and an attR2/attL3 entry vector containing the mCherry coding sequence into the destination vector pK7m34GW (Karimi et al., 2007).

### Transient expression in *N. benthamiana* leaves

Agro‐infiltrations were conducted by infiltrating leaves with equal volumes of agrobacterial suspensions in 10 mm MgCl_2_, 10 mm MES pH 5.6, and 200 μm acetosyringone. Final OD_600_ values were adjusted to 0.6, unless specifically indicated. mGFP5 fluorescence in agro‐infiltrated leaves was imaged either with a high‐intensity handheld UV lamp or with a Typhoon FLA 9000 system (GE Healthcare, www.gehealthcare.com) equipped with a 473 nm excitation laser and a 510–550 nm bandpass filter. Pictures obtained from the Typhoon system were quantified using ImageQuant TL software (GE Healthcare). Confocal imaging was carried out at 4 dpi.

### IP experiments

*Nicotiana benthamiana* leaves (4 dpi) were ground in liquid nitrogen and 0.2 g of tissue powder was resuspended in 1 ml IP buffer (50 mm Tris–HCl pH 7.5, 150 mm NaCl, 10% glycerol, and 0.1% NP40) containing 2 μm MG‐132 and one tablet of cOmplete® protease inhibitor cocktail (Merck Roche, www.sigmaaldrich.com) per 10 ml. Lysates were incubated for 30 min on a rotating wheel and cleared from cell debris twice by centrifugation at 16 000 ***g*** for 10 min. For FHA:P19 IP experiments, cleared lysates were incubated for 30 min with 30 μl of Anti‐HA Magnetic Beads (Pierce, www.thermofisher.com) equilibrated in IP buffer. Bead conjugates were washed three times with IP buffer for 10 min. Immunoprecipitated proteins were retrieved from the beads from 10% of the last wash and resuspended in 1× Western blot loading buffer (10% glycerol, 4% SDS, 62.5 mm Tris–HCl pH 6.8, and 5% 2‐mercaptoethanol). The remaining beads were resuspended in 500 μl of TRI Reagent (Merck, www.sigmaaldrich.com) for RNA extraction. Immunoprecipitated RNA was precipitated from the aqueous phase with the addition of 20 μg of glycogen. RNA from input samples was extracted by adding one volume of Roti‐Phenol/Chloroform/Isoamyl Alcohol (Carl Roth, www.carlroth.com), precipitated from the aqueous phase with one volume of isopropanol in the presence of 0.3 m sodium acetate pH 5.2, and washed with 80% ethanol. For FHA‐GUS IP experiments, cleared lysates were incubated for 30 min on a rotating wheel with 50 μl of Anti‐HA Magnetic Beads (Pierce) equilibrated in IP buffer. Bead conjugates were washed four times with IP buffer for 10 min. Immunoprecipitated proteins were retrieved from 50% of the beads resuspended in 1× Western blot loading buffer (10% glycerol, 4% SDS, 62.5 mm Tris–HCl pH 6.8, and 5% 2‐mercaptoethanol). The remaining beads were resuspended in IP buffer containing 1 mm 4‐MUG (Merck Sigma, www.sigmaaldrich.com) and incubated at 37°C under gentle agitation (600 rpm) for 0, 1, 2, 3, and 5 min. Supernatants were collected and the reactions were stopped by adding 0.2 m Na_2_CO_3_ (Merck Sigma). Technical triplicates of 200 μl were transferred to black flat‐bottom 96‐well plates (Greiner Bio‐One, www.gbo.com) and 4‐MU production was measured with a plate reader (Polarstar Omega, www.bmglabtech.com; excitation: 355 nm, emission: 460–510 nm). A 0.1–10 μm 4‐MU standard curve was used to convert relative fluorescence units (RFU) to nmol 4‐MU. GUS activities (nmol 4‐MU/min) were obtained based on the average of the triplicates after correction using a blank sample and determination of the time‐course trendlines.

### RNA extraction and Northern blot analysis

RNA was extracted from frozen tissues ground in liquid nitrogen using TRI Reagent (Merck) according to the manufacturer's instructions. Equal amounts of RNA (2 μg) or immunoprecipitated RNA fractions were resuspended in 50% formamide and 1× RNA loading buffer (12.5% glycerol, 12.5 mm Tris pH 7.7, 1.25 mM EDTA, and 0.008% bromophenol blue), resolved by electrophoresis on a denaturating polyacrylamide gel (0.5× TBE, 17.5% polyacrylamide/bisacrylamide 19:1, and 8 m urea), transferred to a Hybond‐NX Nylon membrane (Merck Sigma) in 0.5× TBE, and cross‐linked using 1‐ethyl‐3‐(3‐dimethylaminopropyl)carbodiimide according to Pall and Hamilton (2008) for 2 h at 60°C. RNA blots were pre‐hybridized in PerfectHyb™ Plus Hybridization Buffer (Sigma‐Aldrich, www.sigmaaldrich.com) at 42°C for 30 min before adding probes of interest. Random‐radiolabeled probe GFP (Table S1) was generated by incubating PCR fragments, obtained using the *p35S::GFFG* plasmid as PCR template, with the Prime‐a‐Gene® labeling system (Promega, www.promega.com) in the presence of [α‐^32^P]‐dCTP (Hartmann Analytic, www.hartmann‐analytic.de). Oligonucleotide probes (Table S1) were end‐labeled by incubation with T4 PNK (Thermo Fisher Scientific) in the presence of [γ‐^32^P]‐dATP (Hartmann Analytic). Membranes were probed overnight at 42°C and then washed three times with 2× SSC, 2% SDS at 50°C for 15 min and exposed to a storage phosphor screen, followed by imaging on a Typhoon FLA9000 (GE Healthcare). Band quantification was conducted with Image Lab software (Bio‐Rad, www.bio‐rad.com) using auto‐contrasted images. For sequential hybridizations of several probes, membranes were stripped with boiling in 0.1% SDS three times for 15 min before re‐probing.

### RT‐qPCR

Five hundred nanograms of RNA extracted with TRI Reagent was treated with 1 unit of DNAse I (Thermo Fisher Scientific) for 40 min at 37°C and reverse‐transcribed with the Maxima First Strand cDNA Synthesis kit (Thermo Fisher Scientific) according to the manufacturer's instructions. cDNA was diluted 1:3 and 1 μl was used in 10 μl PCR reactions with KAPA SYBR FAST qPCR 2× master mix (Merck Sigma) and gene‐specific primers (0.2 μm each) listed in Table S1. qPCR reactions were performed in triplicate in 384‐well plates using a LightCycler 480 System (Roche, www.diagnostics.roche.com) following the PCR program recommended with the KAPA SYBR FAST qPCR mix. In addition, a melting curve was drawn to verify the specificity of each PCR amplification. Cp values (cycle values of the maximum second derivative of the amplification curves) were calculated for each PCR reaction with LightCycler 480 software. Relative expression values were obtained by calculating 2^−∆Cp^, where ∆Cp represents the difference between the Cp value of the analyzed RNA and the average of the Cp values of *ACTIN* (*ACT*) and *PHYTOENE DESATURASE* (*PDS*), which were used as control mRNAs. Primers for *ACT* and *PDS* were designed based on the sequences from GenBank accession numbers JQ256516.1 and EU165355.1, respectively.

### Western blot

Total proteins were extracted either following the protocol of Hurkman and Tanaka (1986) (Figures 3b, 5b, and 7a) or by adding 4 m urea/100 mm DTT to ground tissues prior to boiling for 5 min (Figure 6c). Proteins were separated by SDS‐PAGE before subsequent transfer onto Western blot membranes. For chemiluminescent detection, proteins were transferred onto Immobilon‐P PVDF membranes (Merck Millipore). Membranes were blocked for 30 min in 1× TBS supplemented with 0.5% non‐fat milk. For FLAG Western blot analysis, membranes were incubated for 1 h at room temperature with monoclonal anti‐FLAG M2‐Peroxidase (HRP) antibody (Sigma, www.sigmaaldrich.com; Table S2). For RFP and GFP Western blot analyses, membranes were incubated with primary antibodies overnight at 4°C (Table S2) and subsequently washed three times with 1× TBS‐T. Membranes were further incubated for 1 h at room temperature with 1:5000 dilutions of HRP‐conjugated goat anti‐rat secondary antibody (Table S2). Protein detection was carried out with Lumi‐Light^PLUS^ Western Blotting ECL Substrate (Roche, www.sigmaaldrich.com) after three washes with 1× TBS‐T and protein bands were imaged with a ChemiDoc Touch Imaging System (Bio‐Rad). Membranes were stained with Coomassie blue to reveal total proteins. For fluorescence detection, proteins were transferred onto Immobilon‐L PVDF membranes (Merck Millipore, www.sigmaaldrich.com). Membranes were blocked for 30 min in 1× TBS supplemented with 0.5% non‐fat milk. Membranes were incubated with a mix of anti‐actin and anti‐GFP primary antibodies overnight at 4°C (Table S2) and subsequently washed three times with 1× TBS‐T. Membranes were further incubated for 1 h at room temperature with dilutions of fluorescent anti‐mouse and anti‐rat secondary antibodies (Table S2) in the dark. Membranes were washed three times with 1× TBS‐T and two times with 1× TBS. Protein detection was carried out on dry membranes by using an Odyssey CLx imaging system (Li‐Cor, www.licor.com) with automatic scanning settings. Protein band signal intensities were quantified using Image Studio Lite software (Li‐Cor).

### Confocal microscopy

Confocal pictures were acquired using a Zeiss LSM 780 microscope (www.zeiss.com/microscopy) controlled by Zeiss Zen software. GFP and mCherry fusion proteins were imaged using a 488 nm excitation laser with detection at 495–550 nm and a 561 nm excitation laser with detection at 575–630 nm, respectively, in sequential acquisition mode. Images were further processed using ImageJ (www.imagej.net).

### Measurements of RFU

*Nicotiana benthamiana* leaves were ground in liquid nitrogen, and 0.5 ml of tissue powder was resuspended in 1 ml of IP buffer (50 mm Tris–HCl pH 7.5, 150 mm NaCl, 10% glycerol, and 0.1% NP40) containing 2 μm MG‐132 and one tablet of cOmplete® protease inhibitor cocktail (Merck Roche) per 10 ml. Lysates were incubated for 30 min on a rotating wheel and then cleared from cell debris by centrifugation at 16 000 ***g*** for 30 min. Protein concentration was determined using the DC Protein Assay (Bio‐Rad), and lysates were adjusted to 250 μg ml^−1^. Technical triplicates of 100 μl were transferred to black flat‐bottom 96‐well plates (Greiner) and fluorescence was measured with a plate reader (Polarstar Omega; excitation: 485 nm, emission: 520 nm). RFU values were obtained as the average of the triplicates after correction using a blank sample and plotted as RFU per μg of protein.

## AUTHOR CONTRIBUTIONS

FJ and FB performed the experiments. FJ, FB, and OV planned and designed the research, analyzed the data, and wrote the manuscript.

## CONFLICT OF INTEREST

A technology employing the P19 protein as an enhancer of transient expression is licensed by Plant Bioscience Limited, Norwich, UK. The corresponding patents list Olivier Voinnet as a co‐inventor.

## Supporting information


**Figure S1.** (a) GFP fluorescence signal at 473 nm of *N. benthamiana* leaves, representative of six independent experiments, 4 days post‐infiltration with the constructs *p35S::FHA:GUS‐Intron* (GUS) and *p35S::GFP5* (GFP) on the left‐hand side, or *p35S::P19* (P19) and *p35S::GFP5* (GFP) on the right‐hand side. (b) Quantification of the GFP fluorescence signal obtained in (a). Black bars: mean. Error bars: standard deviations. *n* = 6.
**Figure S2.** (a) Images of *N. benthamiana* leaves, representative of four independent experiments, taken under UV illumination at 7 days post‐infiltration (dpi) with the constructs *p35S::GFP5* or *pUB10::GFP5*, together with *p35S::FHA:GUS‐Intron* (GUS) or *p35S::P19* (P19). (b) Relative GFP fluorescence units (RFU) measured from the samples depicted in (a). Black bars: mean. Error bars: standard deviations. *n* = 4. (c) *GF* siRNA accumulation in biological duplicates of samples depicted in (a) analyzed by Northern blot using a probe corresponding to the 5′ part of the *GFP* ORF (‘GF’). The probed miR159 and U6 small RNA were used as an endogenous P19 cargo and RNA loading control, respectively. EtBr.: Ethidium bromide staining provides an additional RNA loading control. Black arrows indicate 3′‐end‐trimmed sRNA species. The experiment was independently repeated two times with similar results.
**Figure S3.** (a) Independent biological replicate of experiments presented in Figure 5d. FHA:P19 protein levels in the input, unbound fraction, and IP fraction of *N. benthamiana* leaves at 4 dpi with infiltration medium (−), *p35S::FHA:GUS‐Intron* (GUS), *p35S::FHA:P19* (F:P19), and *p35S::GFFG* (GFFG), as indicated. Coomassie staining of total proteins (Coom.) provides a control for equal loading. (b) Independent biological replicate of experiments presented in Figure 5e. *GF* siRNA levels in samples depicted in (a) analyzed by Northern blot. miR159 and U6 small RNA were used as an endogenous P19 cargo and RNA loading control, respectively. EtBr.: Ethidium bromide staining provides an additional RNA loading control. Black arrows indicate 3′‐end‐trimmed sRNA species.
**Figure S4.** GUS activity in relative MU fluorescence units (RFU) from the FHA:GUS IP fraction of samples depicted in Figure 7c at 0, 1, 2, 3, and 5 min. Plotted: mean. Error bars: standard deviations. *n* = 6.
**Figure S5.** Images of *Nicotiana benthamiana* leaves 4 days post‐infiltration with the constructs *p35S::P19* (P19) and *p35S::ERD2:mCherry* (ERD2), as indicated, under normal light.
**Figure S6.** *p35S::GFP5* expression cassette within the pBI121 binary vector backbone described in Angell and Page (2002) and used in this study.

**Table S1.** Sequences of oligonucleotide primers used in this study.
**Table S2.** List of antibodies used in this study.

## Data Availability

This study includes no data deposited in external repositories. All relevant data can be found within the manuscript and its supporting materials.
