# Supplementary material for: A contemporary reassessment of the enhanced transient expression system based on the tombusviral silencing suppressor protein P19
Source: Plant J. 2022 Dec 14;113(1):186–204. doi: 10.1111/tpj.16032 (PMC10107623; doi:10.1111/tpj.16032)
Supplement: Supplementary file 1 — Figure S1. (a) GFP fluorescence signal at 473 nm of N. benthamiana leaves, representative of six independent experiments, 4 days post‐infiltration with the constructs p35S::FHA:GUS‐Intron (GUS) and p35S::GFP5 (GFP) on the left‐hand side, or p35S::P19 (P19) and p35S::GFP5 (GFP) on the right‐hand side. (b) Quantification of the GFP fluorescence signal obtained in (a). Black bars: mean. Error bars: standard deviations. n = 6. Figure S2. (a) Images of N. benthamiana leaves, representative of four independent experiments, taken under UV illumination at 7 days post‐infiltration (dpi) with the constructs p35S::GFP5 or pUB10::GFP5, together with p35S::FHA:GUS‐Intron (GUS) or p35S::P19 (P19). (b) Relative GFP fluorescence units (RFU) measured from the samples depicted in (a). Black bars: mean. Error bars: standard deviations. n = 4. (c) GF siRNA accumulation in biological duplicates of samples depicted in (a) analyzed by Northern blot using a probe corresponding to the 5′ part of the GFP ORF (‘GF’). The probed miR159 and U6 small RNA were used as an endogenous P19 cargo and RNA loading control, respectively. EtBr.: Ethidium bromide staining provides an additional RNA loading control. Black arrows indicate 3′‐end‐trimmed sRNA species. The experiment was independently repeated two times with similar results. Figure S3. (a) Independent biological replicate of experiments presented in Figure 5d. FHA:P19 protein levels in the input, unbound fraction, and IP fraction of N. benthamiana leaves at 4 dpi with infiltration medium (−), p35S::FHA:GUS‐Intron (GUS), p35S::FHA:P19 (F:P19), and p35S::GFFG (GFFG), as indicated. Coomassie staining of total proteins (Coom.) provides a control for equal loading. (b) Independent biological replicate of experiments presented in Figure 5e. GF siRNA levels in samples depicted in (a) analyzed by Northern blot. miR159 and U6 small RNA were used as an endogenous P19 cargo and RNA loading control, respectively. EtBr.: Ethidium bromide staining provides [file TPJ-113-186-s002.pdf]

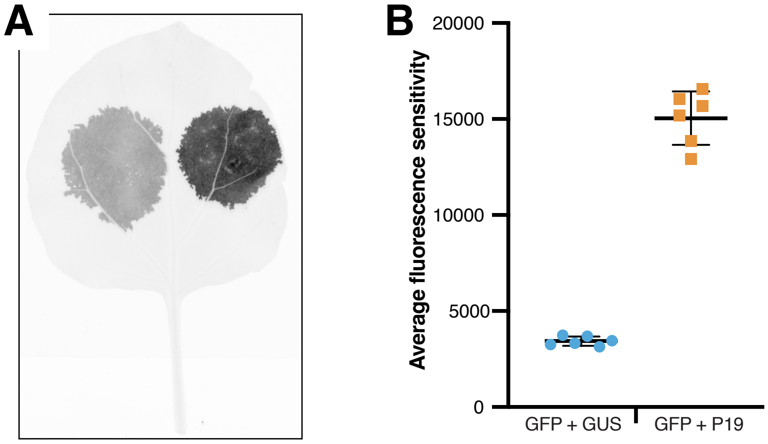

SUPPLEMENTARY FIGURE 1

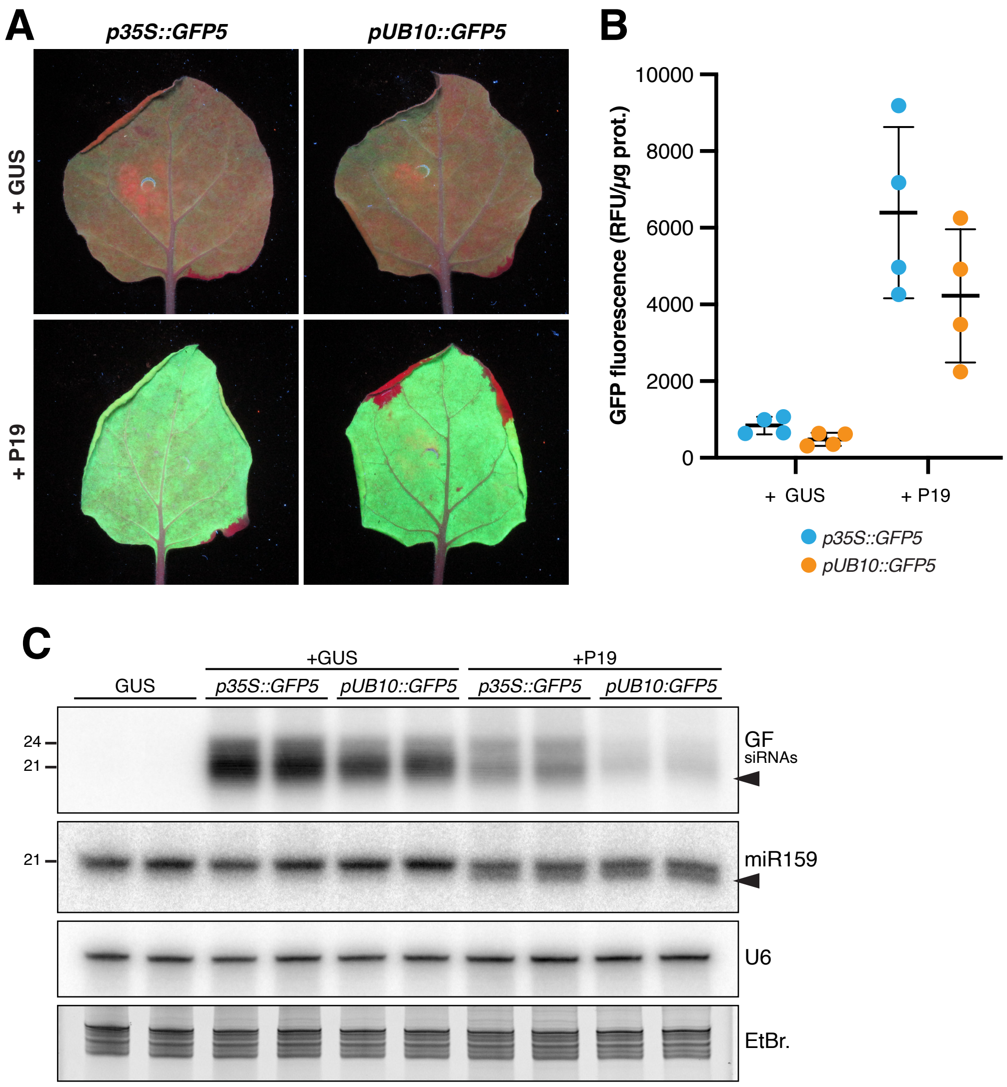

SUPPLEMENTARY FIGURE 2

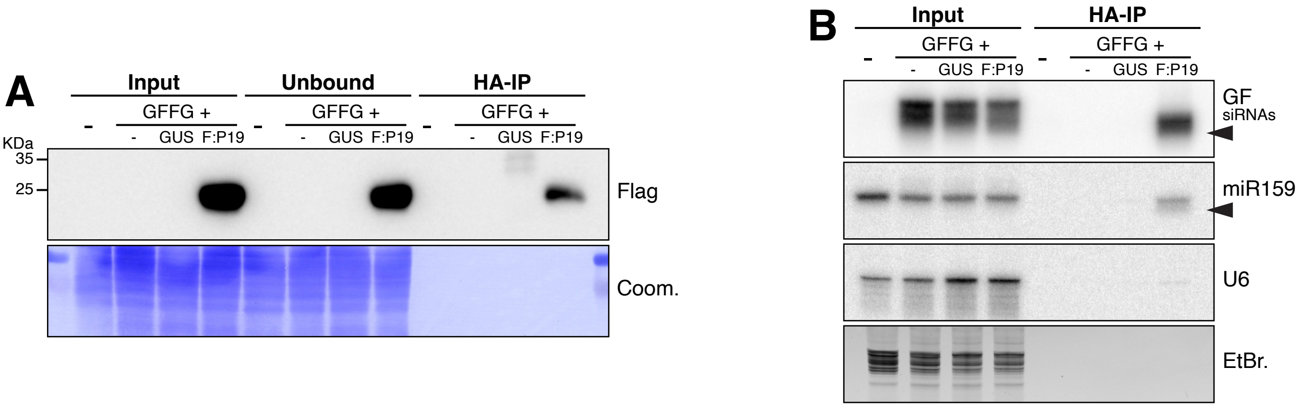

SUPPLEMENTARY FIGURE 3

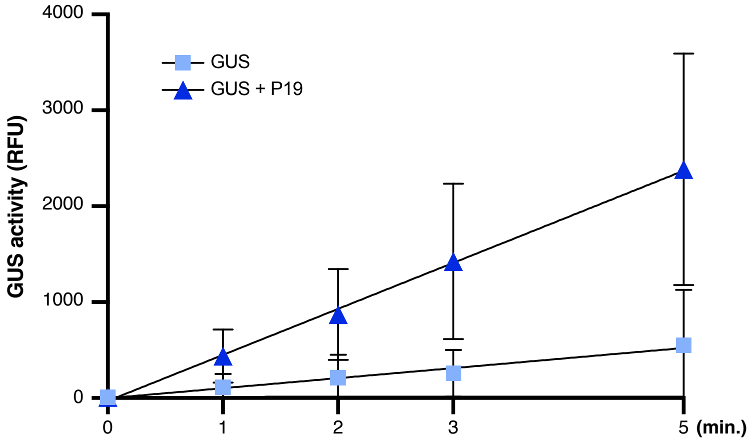

SUPPLEMENTARY FIGURE 4

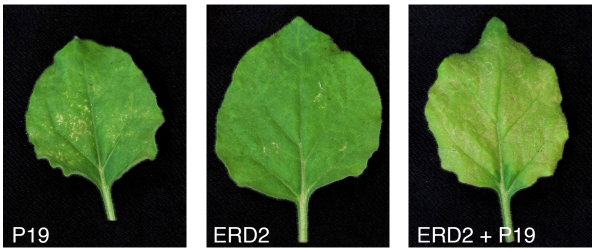

SUPPLEMENTARY FIGURE 5

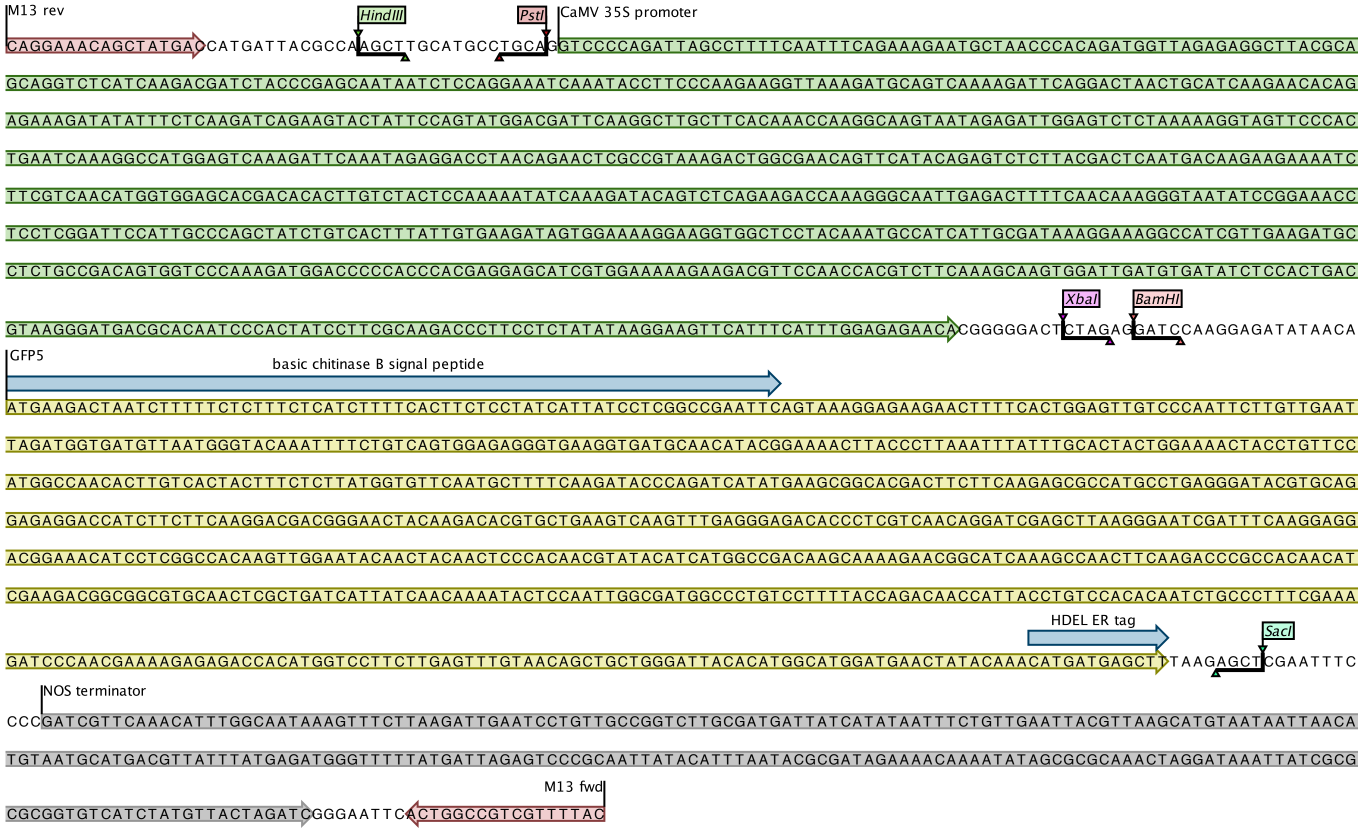

SUPPLEMENTARY FIGURE 6
