# Supplementary material for: A contemporary reassessment of the enhanced transient expression system based on the tombusviral silencing suppressor protein P19
Source: Plant J. 2022 Dec 14;113(1):186–204. doi: 10.1111/tpj.16032 (PMC10107623; doi:10.1111/tpj.16032)
Supplement: Supplementary file 2 — Table S1. Sequences of oligonucleotide primers used in this study. Table S2. List of antibodies used in this study. [file TPJ-113-186-s001.pdf]

SUPPLEMENTARY TABLE 1

Application	Primer name	Sequence
RT-qPCR	NbAct4_For	AGCTATGTATGTTGCTATTCAGG
	NbAct4_Rev	CTAATATCCACATCGCACTTC
	NbPDS_For	TGCCTGAAGACTGGAAAGAG
	NbPDS_Rev	TCCTGATAAGACAGCACCTTC
	GFP_For	ACATCCTCGGCCACAAGTTG
	GFP_Rev	GGTAAAAGGACAGGGCCATC
Probes for small RNA Northern blot	miR159	TAGAGCTCCCTTCAATCCAAA
	U6	AGGGGCCATGCTAATCTTCTC
	GF siRNAs_For	AGTAAAGGAGAAGAACTTTTCACT
	GF siRNAs_Rev	TTCCGTCCTCCTTGAAATCGA
Gateway cloning - BP recombinations	p35S_attB4	GGGGACAACTTTGTATAGAAAAGTTGAAGCTTGCATGCCTGCAGGTC
	p35S_attB1r	GGGGACTGCTTTTTTGTACAAACTTGACTAGTGGATCCTCTAGAGTCCCCCGTGT
	pUB10_attB4	GGGGACAACTTTGTATAGAAAAGTTGCTAGTCTAGCTCAACAGAGCTTTTA
	pUB10_attB1r	GGGGACTGCTTTTTTGTACAAACTTGCCTGTTAATCAGAAAAACTCAGAT
	ACX4_attB1	GGGGACAAGTTTGTACAAAAAAGCAGGCTTAGCGGTGCTTTCATCTGCAGATC
	ACX4_attB2	GGGGACCACTTTGTACAAGAAAGCTGGGTTTAGAGACGGCTACGTGTAG
	GUS_attB2r	GGGGACAGCTTTCTTGTACAAAGTGGCTATGTTACGTCCTGTAGAAACC
	GUS_attB3	GGGGACAACTTTGTATAATAAAGTTGCTCATTGTTTGCCTCCCTGCTG
	GFhp_attB1	GGGGACAAGTTTGTACAAAAAAGCAGGCTTAATGAGTAAAGGAGAAGAACTTTTCA
	GFhp_attB2	GGGGACCACTTTGTACAAGAAAGCTGGGTACGTCCTCCTTGAAATCGATTCCCTT
TOPO Cloning in pENTR_D-TOPO	BRI1_For	CACCATGAAGACTTTTTCAAGCTTC
	BRI1_Rev	TAATTTTCCTTCAGGAACTTC
	ERD2_For	CACCATGAATATCTTTAGATTTGC
	ERD2_Rev	AGCCGGAAGCTTAAGTTTGGTGTTGG
	SNX1_For	CACCATGGAGAGCACGGAGCAGCCG
	SNX1_Rev	GACAGAATAAGAAGCTTCAAG
	VHA-a1_For	CACCATGGAGGAATTCTTAGATAAG
	VHA-a1_Rev	GATTAAAGCGAAAGAGAAAGGC

SUPPLEMENTARY TABLE 2

Antibody name	Product number	Supplier	Incubation conditions	Raised in
Monoclonal ANTI-FLAG® M2-Peroxidase (HRP)	A8592	Sigma	1/8’000 in 1X TBS + 0.5 % milk	Mouse
Monoclonal anti-RFP	5FB	Chromotek	1/5’000 in 1X TBS + 0.5 % milk	Rat
Monoclonal anti-GFP	3H9	Chromotek	1/7’500 in 1X TBS + 0.5 % milk	Rat
Anti-Rat IgG, HRP	7077	Cell Signaling	1/5’000 in 1X TBST + 0.5 % milk	Goat
Monoclonal Anti-actin (plant) antibody	A0480	Sigma	1/8’000 in 1X TBS + 0.5 % milk	Mouse
Anti-Mouse Red IRDye® 680 RD	925-68072	Li-Cor	1/15’000 in 1X TBST + 0.01% SDS + 0.5 % milk	Donkey
Anti-Rat Green IRDye® 800 CW	925-32219	Li-Cor	1/15’000 in 1X TBST + 0.01% SDS + 0.5 % milk	Goat
